# Prospective Case Series of Clinical Signs and Adrenocorticotrophin (ACTH) Concentrations in Seven Horses Transitioning to Pituitary Pars Intermedia Dysfunction (PPID)

**DOI:** 10.3390/vetsci9100572

**Published:** 2022-10-17

**Authors:** Naomi C. Kirkwood, Kristopher J. Hughes, Allison J. Stewart

**Affiliations:** 1 School of Agricultural, Environmental and Veterinary Sciences, Charles Sturt University, Wagga Wagga, NSW 2678, Australia; 2 School of Veterinary Science, Gatton Campus, The University of Queensland, Gatton, QLD 4343, Australia

**Keywords:** endocrine, geriatric, hypertrichosis, immune dysfunction, α-melanocyte stimulating hormone (α-MSH), proopiomelanocortin (POMC) derived peptides, horses, β-endorphin (β-END), thyrotropin-releasing hormone (TRH)

## Abstract

**Simple Summary:**

Pituitary pars intermedia dysfunction (PPID) is a common disease of the geriatric horse population. The most common clinical sign of PPID is hypertrichosis, or a long hair-coat with delayed shedding. Hypertrichosis is the most easily recognized clinical sign of PPID. However, the presence of hypertrichosis is often associated with severe end-stage disease. There is little research investigating sub-clinical or early PPID and the clinical signs associated with these stages of disease. The benefit of being able to recognize early PPID, is that we are able to begin treatment earlier on in disease process, potentially reducing the deleterious consequences of PPID and improving survival. Laboratory tests are available to more accurately diagnose PPID, and these tests include the basal ACTH and TRH-stimulated ACTH tests. Basal ACTH is easy to perform and is recommended in cases where clinical disease is suspected. The TRH-stimulation test improves diagnostic accuracy in early PPID cases. This study documents both test results and clinical signs associated with the transition from subclinical to clinical PPID, so that we are better able to recognize potential early PPID, as well as interpret results in these horses.

**Abstract:**

Poor recognition of subtle clinical abnormalities and equivocal ACTH concentrations make early diagnosis of PPID difficult. Progressive clinical findings and corresponding ACTH concentrations in horses transitioning to PPID over time have not been documented. Seven horses with ACTH concentrations equivocal for PPID (utilizing locally derived, seasonally adjusted diagnostic-cut off values (DCOV)) and no clinical signs of PPID were selected. Sequential measurement of basal and thyrotropin-releasing hormone (TRH)-stimulated ACTH concentrations and recording of clinical findings occurred from October 2017 to November 2021 in a prospective case series. In two horses, marked hypertrichosis developed. Although 1/11 basal ACTH concentrations were below DCOV in 2018, subsequently all basal ACTH concentrations in these two horses without treatment were greater than DCOV. One horse was treated with pergolide which normalized basal ACTH concentrations. Four horses developed intermittent, mild hypertrichosis, and one horse never developed hypertrichosis. Basal ACTH concentrations in these five horses were greater than DCOV in 63/133 (47.4%) of testing points. TRH-stimulated ACTH concentrations in these five horses were greater than DCOV in 77/133 (57.9%) of testing points, sometimes markedly increased and greater than the assay upper limit of detection (LoD) of 1250pg/mL. TRH-stimulated ACTH concentrations were most frequently positive in late summer and early autumn, with 24/37 (64.9%) of TRH-stimulated ACTH concentrations greater than the DCOV in February and March. Horses transitioning to PPID can have subtle clinical signs and equivocal ACTH concentrations. However, TRH-stimulated ACTH concentrations can be markedly greater than DCOV, especially in late summer and early autumn (February and March) allowing for identification of subclinical and transitional cases.

## 1. Introduction

Pituitary pars intermedia dysfunction (PPID) is a disease of aged horses, affecting 20–25% of horses over the age of 15 years [1,2,3,4,5]. Pituitary pars intermedia dysfunction is caused by loss of dopaminergic inhibition on the melanotropes of the pituitary pars intermedia (PI) due to oxidative-stress and subsequent neurodegeneration of dopaminergic neurons within the hypothalamus [6,7,8]. The loss of dopaminergic inhibition results in hyperplasia, microadenoma or macroadenoma formation of the pars intermedia, and overproduction of POMC derived peptides including ACTH, α-MSH, β-END and corticotropin like intermediate lobe peptide (CLIP) [4,9,10,11,12,13].The presence of clinical signs such as hypertrichosis, lethargy, muscle atrophy, pendulous abdomen, polydipsia/polyuria (PU/PD), hyperhidrosis, anhidrosis, and recurrent infections all raise suspicion of PPID [3,14,15,16,17]. However, clinical signs in horses with early disease can be subtle, such as delayed hair shedding, or non-specific findings including loss of top-line, lethargy, poor performance and infertility can be present [4,5,14]. Subclinical disease can also occur [4,5]. 

A presumptive diagnosis of PPID can be made based on pathognomonic hypertrichosis in aged horses with marked disease [15,18,19,20,21]. Early detection of disease and initiation of treatment is associated with increased short-term survival [14]. Laboratory testing is recommended in cases where PPID is suspected [22], to assess response to treatment and to periodically monitor for disease progression [22,23]. However, currently there is no perfect ante-mortem diagnostic test for the accurate diagnosis of early PPID [24,25]. PPID can have a severe impact on quality and duration of life. The ability to diagnose and begin treatment prior to development of severe clinical signs such as thermoregulatory disturbances, immune dysfunction, severe weight loss and laminitis may be beneficial [5,14,17]. In horses with obvious clinical disease, the current recommendation for diagnosis is to assess the basal plasma concentrations of ACTH utilising seasonally adjusted diagnostic thresholds [22]. For diagnosis in horses with few, mild or early clinical signs, or equivocal basal ACTH concentrations, a TRH-stimulation test should be performed due to increased diagnostic accuracy for the detection of PPID [2,17,20,26]. Exogenous administration if TRH will lead to binding of TRH receptors on the melanotrophs of the pars intermedia and increase secretion of POMC derived peptides [27]. Seasonally adjusted diagnostic thresholds must be used to interpret all results, as basal and TRH-stimulated ACTH follows a circannual rhythm with ACTH concentrations peaking in early autumn [2,23,28,29,30]. Failure to utilise seasonally adjusted diagnostic thresholds can result in false positive, or false negative results [2]. Plasma concentrations of ACTH can be increased with stress, illness, exercise diet, season, breed and age [29,31,32,33,34,35], and interpretation of test results should take these factors into consideration. Other diagnostic techniques for diagnosis of PPID include measurement of endogenous α-MSH concentrations, which is not commercially available [24], and the overnight dexamethasone suppression test. Seasonally adjusted DCOV are yet to be established for this diagnostic test [23,24,28]. 

Poor recognition of subtle clinical signs and equivocal ACTH concentrations makes early diagnosis of PPID in horses challenging. Development of clinical abnormalities and corresponding basal and TRH-stimulated ACTH concentrations in horses transitioning to PPID over time have not been documented. Therefore, the aim of this study was to document the clinical signs and endocrinological changes associated with the transition from subclinical to clinical PPID. The study will improve recognition of subtle clinical signs and emphasize the need for repeated testing in horses with equivocal basal ACTH concentrations. This will lead to earlier diagnosis and initiation of treatment, potentially improving quality of life for PPID affected horses. 

## 2. Materials and Methods

### 2.1. Study Design

One hundred and thirteen light-breed horses underwent basal and TRH stimulated ACTH tests every month over a 12 month period in a study to determine seasonal DCOV and reference ranges and is published elsewhere [2]. In brief, horses were allocated to either a PPID group (n = 34), a normal group (n = 72) or an equivocal group. Horses were allocated to the PPID group based on clinical signs, necropsy findings or recurrent plasma baseline or post-TRH ACTH concentrations considered as outliers by robust regression and outlier removal (ROUT) method with Q = 1% in ≥ 6 of the 12 months of testing [36]. Horses in the normal group had baseline or TRH-stimulated ACTH concentrations not considered as an outlier by ROUT method with Q = 1% identified in the ACTH results in ≤ 2 out of 12 months tested [2]. Horses with no clinical signs of PPID, but with outlier results for ≥ 3 out of the 12 months tested were suspected of having subclinical PPID and were recruited into the current study. Basal ACTH and TRH-stimulated ACTH concentrations of the selected horses were measured repeatedly from October 2017 to November 2021.

At each sampling point, horses were restrained in their home paddock with a headcollar and lead rope. A physical examination was performed, and photographs were taken of each horse to document the development of hypertrichosis. A hypertrichosis score from 0–5 (Table 1) was recorded and approved by all three authors. Basal ACTH and TRH-stimulation tests were performed at the time of physical examination in the paddock. No horses travelled in the 24 h prior to sampling. All horses received the same free choice pasture and supplemental hay when required, and water ad libitum. 

### 2.2. Blood Sample Collection and Analysis

Two blood samples immediately before and 30 minutes after intravenous administration of 1 mg of TRH ((Sigma-Aldrich Pty Ltd. [subsidiary of Merck], North Ryde BC, Sydney, Australia) [2,26,37,38,39,40] were collected for subsequent measurement of basal and TRH-stimulated ACTH concentrations. Although a 10 min post-TRH sampling time is often considered more convenient for equine practitioners, the 30 min post-TRH sampling time was selected as this sampling time is considered to be less impacted by delays of up to 30 s [41].

Blood samples were obtained from a jugular vein and collected in plastic EDTA tubes (BD, Belliver Industrial Estate, Plymouth, UK), kept on ice, centrifuged and plasma separated within 2 h of sample collection. Analysis of plasma ACTH concentrations were measured within 4 hours using a chemiluminescent immunoassay immunoassay (Immulite 1000 Chemiluminescent Assay, Siemens, Bayswater, Victoria, Australia) with an interassay coefficient of variation of 4.8% and an intraassay coefficient of variation of 5.4% as previously described [38,40]. 

Samples were analysed on the University of Queensland Gatton campus (Veterinary Laboratory Services). The protocol was approved by the Institutional Animal Ethics Committee (ethics approval code: 562/18). 

### 2.3. Data Analysis

Basal and TRH-stimulated ACTH concentrations were graphed using JMP Version 16.1.0 (Copyright 2020–2021 SAS Institute, Cary, NC, USA.). Monthly DCOV established in a recent study [2] conducted in the same geographical location were graphed against the monthly basal and TRH-stimulated ACTH concentrations obtained from the seven horses included in the study. This provided a visual comparison of the difference between the ACTH concentrations of the 7 individual horses in this case series and the referenced monthly DCOV.

## 3. Results

Two horses were subjected to euthanasia due to health issues (Horse 1 and Horse 7). In two horses (Horse 2 and Horse 4), marked hypertrichosis developed. Basal and TRH-stimulated ACTH concentrations in these horses were greater than DCOV in 45/47 testing points (95.7%). Four horses developed intermittent, mild hypertrichosis, and one horse never developed hypertrichosis. Basal ACTH concentrations in these five horses were greater than DCOV in 63/133 (47.4%) testing points. Basal ACTH concentrations were most frequently increased in late summer and early autumn, with 19/37 (51.4%) of basal ACTH concentrations greater than the DCOV in February and March compared to 44/96 (45.8%) between April and January. TRH-stimulated ACTH concentrations in these five horses were greater than DCOV in 77/133 (57.9%) testing points and sometimes markedly increased to above the assay upper LoD of 1250 pg/mL [16,42,43]. TRH-stimulated ACTH concentrations were most frequently greater than the DCOV in late summer and early autumn, with 24/37 (64.9%) of TRH-stimulated ACTH concentrations greater than the DCOV in February and March compared to 53/96 (55.2%) between April and January.

### 3.1. Horse 1

**Figure 1:**
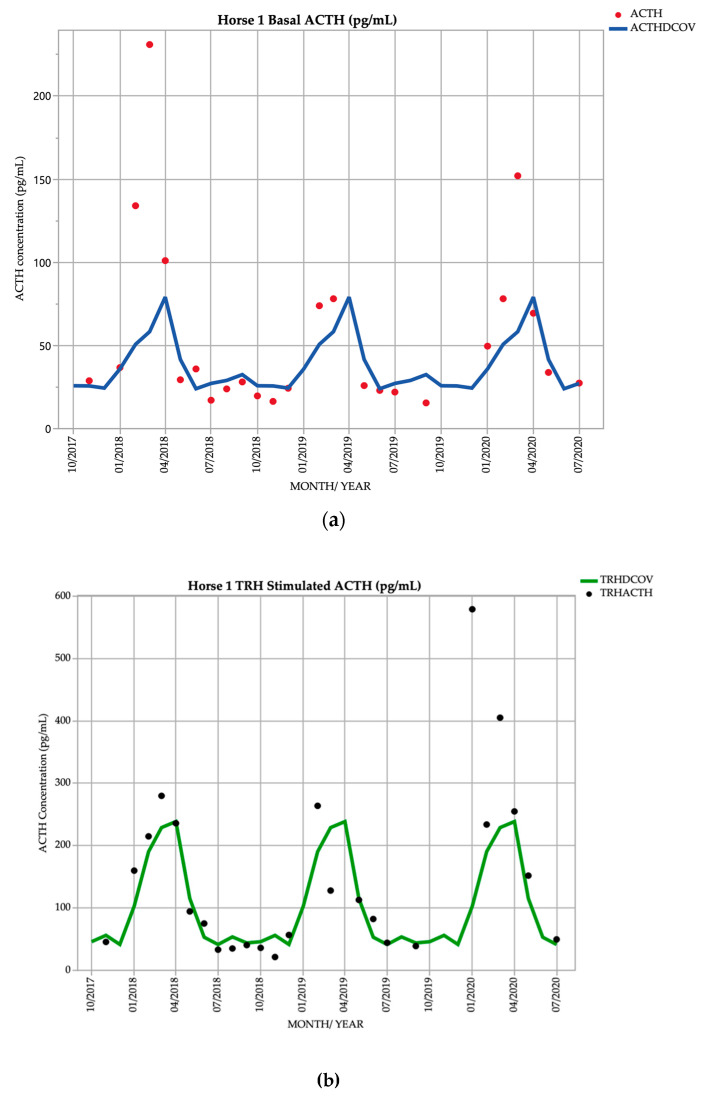
Monthly ACTH concentration (pg/mL) in Horse 1 (26-year-old Stockhorse mare), compared to locally derived, seasonally adjusted DCOV [2]. (**a**) Basal ACTH concentrations (red points) and basal ACTH DCOV (blue line) (**b**) TRH-stimulated ACTH concentrations (black points) and TRH-stimulated ACTH DCOV (green line).

Horse 1, a 26-year-old Stockhorse mare, demonstrated basal ACTH concentrations ≥ DCOV in 12/25 (48.0%) of testing points, and TRH-stimulated ACTH concentrations ≥ DCOV in 15/25 (60.0%) of testing points. Basal ACTH concentrations were most frequently ≥ DCOV in late summer and early autumn (February and March) with 6/6 (100.0%) of basal ACTH concentrations ≥ DCOV. TRH-stimulated ACTH concentrations were most frequently ≥ DCOV in late summer and early autumn (February and March) with 5/6 (83.3%) of TRH-stimulated ACTH concentrations ≥ DCOV (Figure 1). Mild hypertrichosis (1/5) (Table 1) with delayed shedding was not observed until September 2019, two years after the detection of late summer/autumnal increases in basal and TRH stimulated ACTH concentrations (Table 2). 

Horse 1 demonstrated episodes of respiratory distress, nostril flaring and tachypnoea (Video S1) in March 2019 and January 2020 that may have been subsequent to PPID-associated thermodysregulation. Results of haematological, serum biochemical and thoracic radiographic and ultrasonographic examinations were unremarkable. Cytological examination of a bronchioalveolar lavage sample revealed mild chronic inflammation, but no evidence of equine asthma, bacterial infection, or haemorrhage. The episodes were also not accompanied by pyrexia. In July 2020 the mare was subjected to euthanasia due to severe colic (mesenteric rent causing small intestinal strangulation and partial large colon torsion). Post-mortem assessment of the pituitary gland was not performed. 

### 3.2. Horse 2

**Figure 2:**
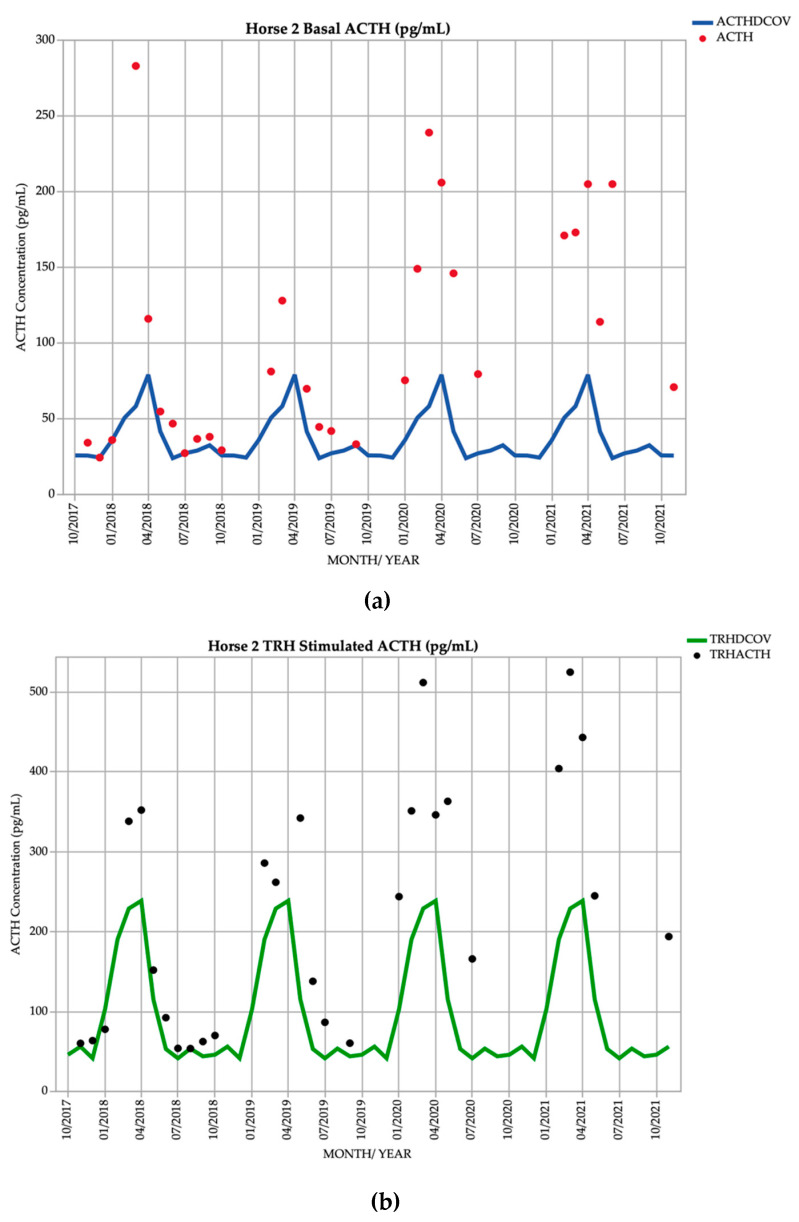
Monthly ACTH concentration (pg/mL) in Horse 2 (21-year-old Warmblood mare), compared to locally derived, seasonally adjusted DCOV [2]. (**a**) Basal ACTH concentrations (red points) and basal ACTH DCOV (blue line) (**b**) TRH-stimulated ACTH concentrations (black points) and TRH-stimulated ACTH DCOV (green line).

Horse 2, a 21-year-old Warmblood mare, demonstrated basal ACTH concentrations ≥ DCOV in 29/29 (100%) of testing points, and TRH-stimulated ACTH concentrations ≥ DCOV in 27/28 (96.4%) of testing points. TRH-stimulated ACTH concentrations were most frequently ≥ DCOV in late summer and early autumn (February and March) with 7/7 (100%) of TRH-stimulated ACTH concentrations ≥ DCOV (Figure 2). This horse had a hypertrichosis score of ≥ 1/5 (Table 1) at every testing point except in February 2019 when with a diagnosis of subclinical PPID was made (Table 2). Hypertrichosis in this horse was frequently marked, with a score of 5/5 (Table 1) in September 2019 and September 2020 (Table 2). 

In August 2021 and February 2022 Horse 2 developed recurrent metritis. This horse lost bodyweight towards the end of the study despite increased feed supplementation. The mare was subjected to euthanasia in June 2022 due to the metritis and weight loss. The pituitary gland was grossly enlarged (histopathology pending). Final basal and TRH stimulated ACTH concentrations were 97 pg/mL and 283 pg/mL, respectively. 

### 3.3. Horse 3

**Figure 3:**
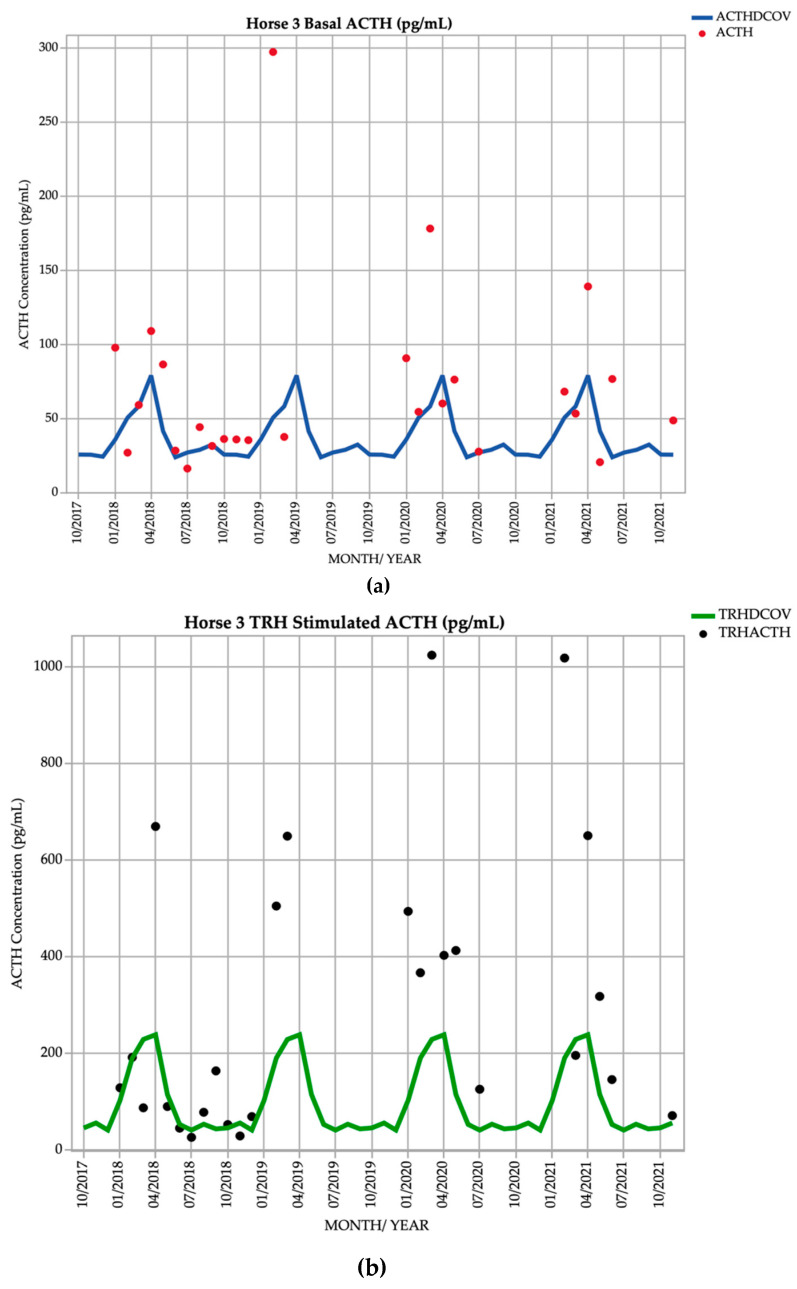
Monthly ACTH concentration (pg/mL) in Horse 3 (12-year-old Standardbred gelding), compared to locally derived, seasonally adjusted DCOV [2]. (**a**) Basal ACTH concentrations (red points) and basal ACTH DCOV (blue line) (**b**) TRH-stimulated ACTH concentrations (black points) and TRH-stimulated ACTH DCOV (green line).

Horse 3, a 14-year-old Standardbred gelding, demonstrated basal ACTH concentrations ≥ DCOV in 20/26 (76.9%) of testing points, and TRH-stimulated ACTH concentrations ≥ DCOV in 20/26 (76.9%) of testing points. Basal ACTH concentrations were most frequently ≥ DCOV in winter, spring and early summer (June to January) with 11/12 (91.6%) of basal ACTH concentrations ≥ DCOV. TRH-stimulated ACTH concentrations were most frequently ≥ DCOV in late summer and autumn (February and March) with 11/14 (78.6%) of TRH-stimulated ACTH concentrations ≥ DCOV (Figure 3). This horse had no abnormal clinical signs on most examinations representing subclinical PPID. Mild hypertrichosis (1/5) (Table 1) was observed in March 2019, September 2020, October 2021 and November 2021, and delayed shedding in the spring months. The presence of hypertrichosis was minimal and intermittent, while 9/12 (75%) of basal and 11/12 (91.7%) of post TRH ACTH concentrations were increased after January 2020 (Table 2). 

Horse 3 suffered from superficial corneal ulceration in May 2020. The ulcer responded well to treatment and did not reoccur, no additional clinical signs of PPID were noted.

### 3.4. Horse 4

**Figure 4:**
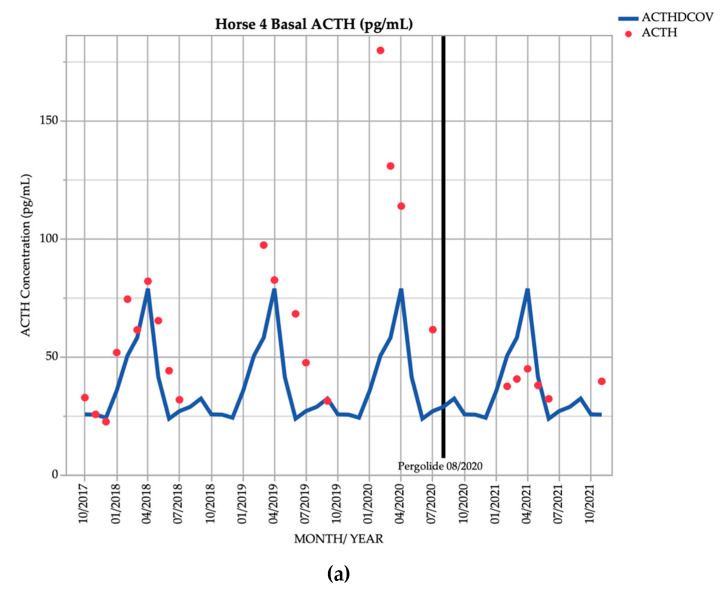

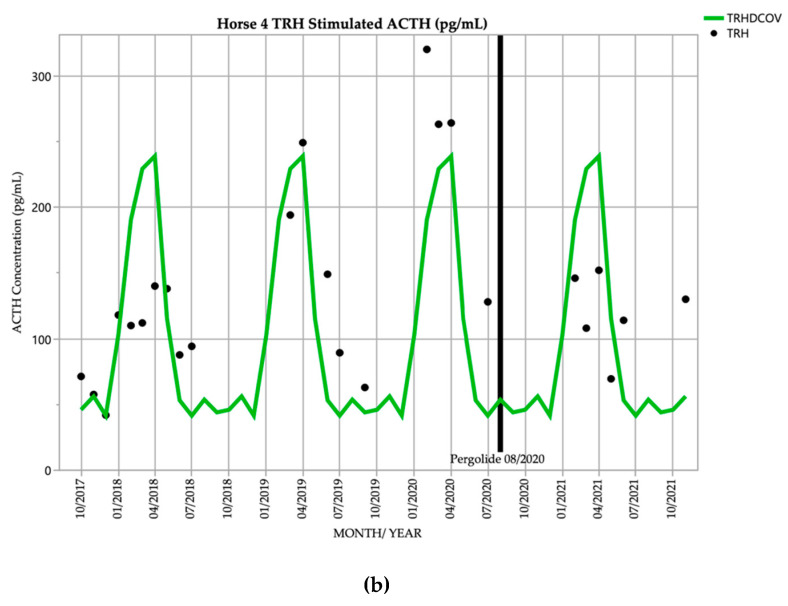
Monthly ACTH concentration (pg/mL) in Horse 4 (21-year-old Stockhorse gelding), compared to locally derived, seasonally adjusted DCOV [2]. (**a**) Basal ACTH concentrations (red points) and basal ACTH DCOV (blue line) (**b**) TRH-stimulated ACTH concentrations (black points) and TRH-stimulated ACTH DCOV (green line).

Horse 4, a 21-year-old Stockhorse gelding, demonstrated basal ACTH concentrations ≥ DCOV in 16/18 (88.9%) of testing points, and TRH-stimulated ACTH concentrations ≥ DCOV in 14/18 (77.8%) of testing points prior to treatment with pergolide. After February 2019, all basal and TRH stimulated ACTH concentrations were ≥ DCOV prior to treatment with pergolide (Figure 4). This horse demonstrated hypertrichosis at all testing points except in February 2019, with marked hypertrichosis (5/5) (Table 1) in September 2020 at commencement of treatment, and less marked hypertrichosis following treatment (Table 2). Delayed shedding was noticed each spring. 

In September 2020 and March 2021, this horse demonstrated episodes of severe tachypnoea, mild tachycardia, and pyrexia. Results of thoracic and abdominal ultrasonographic, haematological, serum biochemical examination and abdominocentesis were unremarkable, and the episodes resolved after administration of 1.1 mg/kg flunixin meglumine IV. Episodes appeared to be due to heat stress, possibly from thermoregulatory disturbances attributed to PPID, and did not reoccur after treatment with pergolide and reduction in hypertrichosis severity.

### 3.5. Horse 5

**Figure 5:**
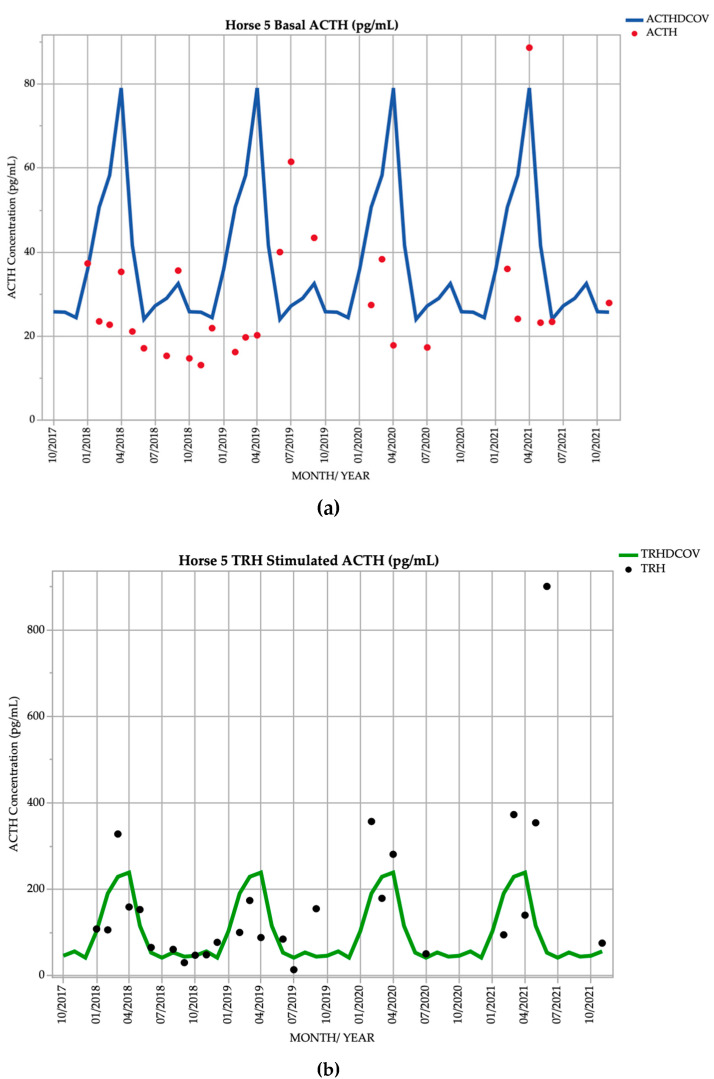
Monthly ACTH concentration (pg/mL) in Horse 5 (21-year-old Standardbred mare), compared to locally derived, seasonally adjusted DCOV [2]. (**a**) Basal ACTH concentrations (red points) and basal ACTH DCOV (blue line) (**b**) TRH-stimulated ACTH concentrations (black points) and TRH-stimulated ACTH DCOV (green line).

Horse 5, a 22yo Standardbred mare, demonstrated basal ACTH concentrations ≥ DCOV in 7/27 (25.9%) of testing points, and TRH-stimulated ACTH concentrations ≥ DCOV in 16/27 (59.3%) of testing points (Figure 5). This horse did not demonstrate hypertrichosis or delayed shedding at any point throughout the study (Table 2).

Horse 5 suffered from recurrent corneal ulceration and suspected viral keratitis from September to November 2020. The condition resolved after a few months of treatment with chloramphenicol, ofloxacin, idoxuridine, atropine and phenylbutazone. 

### 3.6. Horse 6

**Figure 6:**
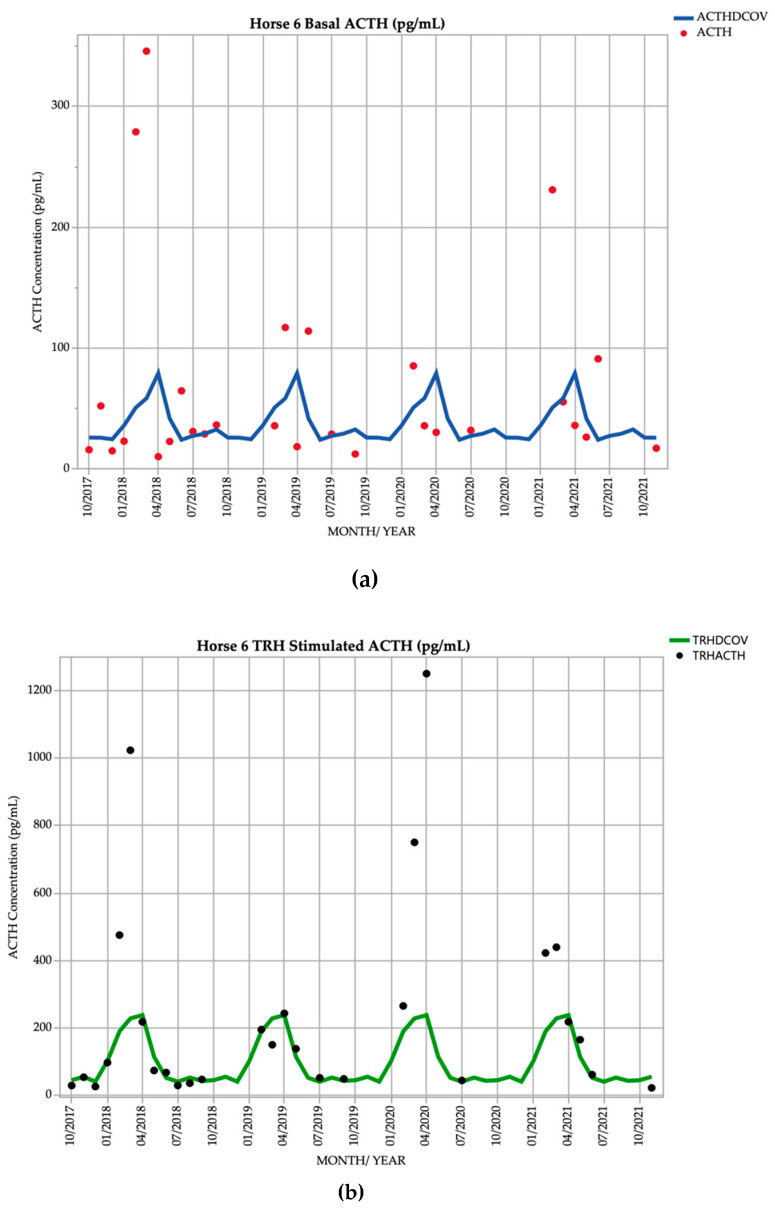
Monthly ACTH concentration (pg/mL) in Horse 6 (16-year-old Standardbred mare), compared to locally derived, seasonally adjusted DCOV [2]. (**a**) Basal ACTH concentrations (red points) and basal ACTH DCOV (blue line) (**b**) TRH-stimulated ACTH concentrations (black points) and TRH-stimulated ACTH DCOV (green line).

Horse 6, a 16-year-old Standardbred mare demonstrated basal ACTH concentrations ≥ DCOV in 13/27 (48.1%) of testing points, and TRH-stimulated ACTH concentrations ≥ DCOV in 17/27 (63.0%) of testing points. Basal ACTH concentrations were most frequently ≥ DCOV in late summer and early autumn (February and March) with 5/8 (62.5%) of basal ACTH concentrations ≥ DCOV. TRH-stimulated ACTH concentrations were most frequently ≥ DCOV in late summer and early autumn (February and March) with 7/8 (87.5%) of TRH-stimulated ACTH concentrations ≥ DCOV (Figure 6). Delayed shedding and mild hypertrichosis (1/5) (Table 1) was present for the first time in September 2021 (Table 2), and no other clinical signs of PPID were noted; therefore, the mare was subclinical for PPID for the majority of the study.

### 3.7. Horse 7

**Figure 7:**
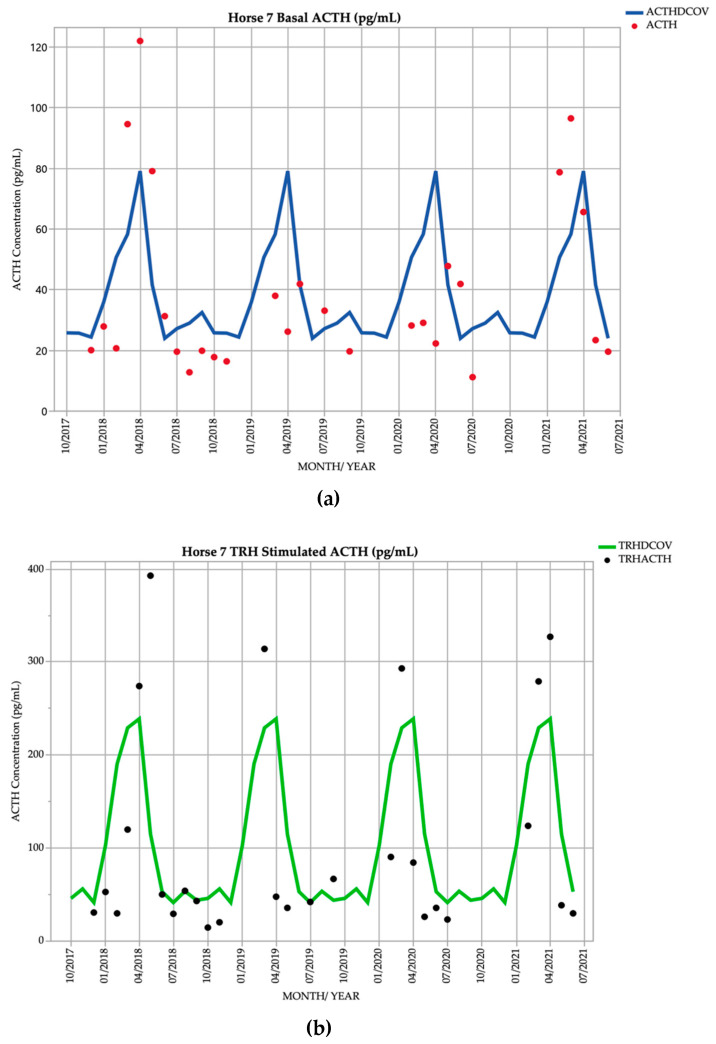
Monthly ACTH concentration (pg/mL) in Horse 7 (18-year-old Standardbred gelding), compared to locally derived, seasonally adjusted DCOV [2]. (**a**) Basal ACTH concentrations (red points) and basal ACTH DCOV (blue line) (**b**) TRH-stimulated ACTH concentrations (black points) and TRH-stimulated ACTH DCOV (green line).

Horse 7, an 18-year-old Standardbred gelding, demonstrated basal ACTH concentrations ≥ DCOV in 11/28 (39.3%) of testing points, and TRH-stimulated ACTH concentrations ≥ DCOV in 9/28 (32.1%) of testing points. Basal ACTH concentrations were most frequently ≥ DCOV in autumn with 7/12 (58.3%) of basal ACTH concentrations ≥ DCOV. TRH-stimulated ACTH concentrations were most frequently ≥ DCOV in autumn with 6/12 (50.0%) of TRH-stimulated ACTH concentrations ≥ DCOV (Figure 7). Delayed shedding and mild hypertrichosis (1/5) (Table 1) were noted in September 2019 (Table 2). Subclinical PPID could consistently be diagnosed by the TRH stimulation test in the autumn. 

In July 2021, Horse 7 was subjected to euthanasia due to tooth root abscessation. A necropsy was performed which revealed mild, diffuse hyperplasia of the PI (grade 2) consistent with PPID [16], and mild chronic multifocal fibrosis affecting 60% of all lung lobes. This gelding also suffered from recurrent corneal ulceration and provisional diagnosis of viral keratitis with episodes in March 2020, February 2021. The horse was treated with acyclovir, idoxuridine, triple ophthalmologic antibiotic ointment (zinc bacitracin, neomycin sulphate and polymyxin B sulphate), atropine and phenylbutazone and responded after long-term treatment.

## 4. Discussion

The main results of the study are that in the early stages of PPID (a), clinical signs can be subtle or absent; (b) basal ACTH concentrations are variable between months and frequently equivocal; (c) increases in ACTH concentrations especially in the autumn can be markedly increased prior to the development of hypertrichosis; (d) TRH-stimulation testing in late summer or autumn may be useful for identification of most transitional cases. 

Clinical signs of PPID are well documented [3,4,15,26,44]. Hypertrichosis is one of the most frequently recognised clinical signs associated with PPID and is pathognomonic for the disease [4,45,46]. In other studies, hypertrichosis was reported to be the most prevalent clinical sign associated with the disease [3,4,47,48]. Therefore, presence of hypertrichosis was graded throughout this study. However, assessing the severity hypertrichosis is subjective. To increase the objective nature of assessment of hypertrichosis, a novel ordinal scale from 0–5 was developed (Table 1). In the early stages of disease, horses may only display subtle clinical signs such as delayed hair shedding, which may not be detected by owners and veterinarians [4]. In this study, horses transitioning to PPID frequently displayed subtle or no clinical signs. Six of the seven horses included in the study demonstrated delayed shedding in the spring months. Of these horses, four did not display any hypertrichosis for two years after subclinical PPID was identified. As the disease progressed, hypertrichosis became more marked. Horses that demonstrate mild hypertrichosis do not always have basal or TRH-stimulated ACTH concentrations greater than the DCOV. To detect PPID in the early stages of disease, clinical signs alone should not be relied upon for diagnosis. 

While hypertrichosis is pathognomonic for PPID, subtle changes such as recurrent infections, delayed healing, or disrupted thermoregulation and heat stress should also raise suspicion of subclinical PPID [44,49,50,51,52,53]. Opportunistic infections occur in 35% of horses with PPID, compared to only 11% of aged horses that do not have the disease [44]. Impaired neutrophil chemotaxis and function has been demonstrated in PPID affected horses [49]. A recent study found that horses with PPID also have a lower lymphocyte count, and decreased interferon γ production from peripheral blood mononuclear cells (PBMCs) after stimulation with *Rhodococcus equi* and *Escherichia coli* [54]. However, the same study also found that PPID and control horses produced similar immune responses to PMA/ionomycin stimulation [54]. These findings suggest altered systemic immune function in horses with PPID increasing susceptibility to opportunistic infections specifically [54,55]. In this study, Horse 2 had recurrent metritis, Horses 5 and 7 suffered from possible recurrent viral keratitis, and Horse 7 had tooth root abscessation, which may have been influenced by secondary immune dysfunction associated with PPID. Horses with PPID have increased cortisol concentrations in tears compared to healthy counterparts, as well as reduced corneal sensitivity which may contribute to delayed corneal wound healing [50,56]. In one study of horses with PPID, the prevalence of corneal ulceration was 18.2%, and keratitis is one of the most common sites of infection in horses with PPID [3,57]. 

Anhidrosis and heat stress with secondary exercise intolerance are also clinical features of PPID [51]. Horses in warmer and more humid climates closer to the equator tend to have a higher frequency of hyperhidrosis and anhidrosis than in cooler, more temperate, climates [14,51]. Anhidrosis results when sweat glands become exhausted. The exact pathophysiology of hyperhidrosis and anhidrosis in horses with PPID has not been established but plasma ACTH concentrations correlate more strongly to sweat production than α-MSH [53]. Horse 1 and Horse 4 both demonstrated episodes of heat stress with characteristic tachypnoea, nostril flare, and tachycardia. Only one horse was profoundly hyperthermic. However, with no abnormalities detected on thoracic or abdominal ultrasonography, abdominocentesis, haematology, biochemistry, thoracic radiographs or BAL, the most likely cause of these episodes was considered to be heat stress.

In the early stages of PPID, basal ACTH concentrations are frequently equivocal for the disease [22]. When clinical signs are indistinct, determination of TRH-stimulated ACTH concentration is useful for increased sensitivity of diagnosis [1,2,17,26]. In this study, 47.4% of basal ACTH concentrations in horses with subtle clinical signs (Horse 1, Horse 3, Horse 5, Horse 6 and Horse 7) were greater than the DCOV. TRH-stimulated ACTH concentrations in these horses were more frequently increased, with 57.9% of TRH-stimulated ACTH concentrations greater than DCOV. TRH-stimulated ACTH concentration was more useful for diagnosis of PPID in this study, but sensitivity was not established as this was a descriptive study with a small sample size. Horse 2 and Horse 4 were more severely affected by PPID, with consistently marked hypertrichosis (Table 2). In these horses, there was little difference between the diagnostic accuracy of basal and TRH-stimulated ACTH concentrations (Figure 2 and Figure 4). In these two horses that developed hypertrichosis in the first year of the study, 95.7% of basal ACTH concentrations were greater than the DCOV. TRH-stimulated ACTH concentrations in these horses were greater than the DCOV in 89.4% of testing points. In these horses, TRH-stimulation tests had slightly reduced accuracy for detection of PPID compared to basal ACTH (Figure 2 and Figure 4). However, this was likely due to a small sample size and individual hypothalamic dopamine production and requires further research.

Currently, using the TRH-stimulation test in autumn is not recommended for diagnosis of PPID [17,22,58]. However, the results of recent studies suggest that while variation in TRH-stimulated ACTH concentrations is greater in autumn, diagnostic accuracy should not be impacted, providing monthly DCOV and RI are utilized [2,41]. Over the current study period, 57.9% of TRH-stimulated ACTH concentrations were greater than the DCOV, whereas TRH-stimulation testing in late summer or autumn (February or March) detected 64.9% of ACTH concentrations greater than the DCOV. For most horses, increased diagnostic accuracy of both basal and TRH-stimulated ACTH tests was demonstrated in the autumn, with the TRH-stimulation test identifying most transitional cases in late summer and autumn. Further research could be conducted in a larger sample of horses transitioning to PPID to determine sensitivity and specificity of the TRH-stimulation test in different seasons of the year.

While the TRH-stimulation test is recommended for use in subclinical cases of PPID, its use is limited in some countries. TRH is available as a compounded product in the USA, but in Australia and Europe, TRH is only available as a chemical grade reagent, generally limiting its use to referral hospitals [39,41,59]. In cases where TRH is unavailable, basal ACTH should be used for diagnosis. Basal ACTH was thought to be the most sensitive and specific diagnostic test in the autumn due to increased differences in ACTH concentration between affected and unaffected horses [60]. More recently, the accuracy of basal ACTH concentrations has been shown to be reduced in autumn [2]. In this study, 47.4% of basal ACTH concentrations taken from horses transitioning to PPID in winter, spring or summer were greater than the DCOV. In autumn, 51.4% of basal ACTH concentrations were greater than the DCOV. If TRH-stimulation testing is not possible, basal ACTH can be used and interpreted in accordance with seasonally DCOV. If basal ACTH concentration is within normal limits, testing should be repeated in 3-6 months if PPID is suspected based on clinical signs [22]. The results from this study show that during the transitional period some results may be positive and some negative, but over time, as the disease becomes clinical, then the laboratory testing is more likely to be positive.

It has been well established that in the early stages of PPID, ACTH concentrations can be equivocal for the diagnosis of PPID [22]. However, this study demonstrated that horses with few to no clinical signs of PPID can have occasional marked increases in ACTH concentrations, which previously has not been demonstrated. Horses 1, 3, 5, 6 and 7 demonstrated few to no clinical signs of PPID throughout the study. However, basal ACTH concentrations in these horses reached values as high as 346 pg/mL (Figure 6a), and TRH-stimulated ACTH concentrations exceeding the assay LoD of 1250 pg/mL [16,42,43] (Figure 6b). To avoid false positive test results and erroneous clinical-decision making, including commencement of un-necessary pergolide treatment, it has been recommended that clinically normal horses should not undergo basal ACTH concentration testing [22]. However, if the owner wishes to avoid the deleterious consequences of clinical PPID (such as muscle loss and increased risk of infections), then identification of sub-clinically affected horses by detection of repeatedly elevated ACTH concentrations and judicious prophylactic treatment with pergolide may be warranted. Although many experts do not recommend testing horses in absence of clinical signs [22], this study demonstrates that subclinical disease can be identified and horses will subsequently transition to clinical PPID within 1-3 years. Detection of disease in the subclinical stages and early initiation of treatment is associated with increased survival [26], and treatment with pergolide reduces clinical signs [46,53,61,62,63,64]. Treating PPID prior to the onset of clinical signs that may be performance limiting, or potentially predispose to clinical abnormalities (infections such as keratitis, tooth root abscesses, metritis or chronic hoof abscessation) could improve quality of life and increase survival. 

Previously, the systemic effects of PPID and the impact on quality of life for affected horses have likely been underestimated. More recent literature suggests that laboratory testing is recommended in cases where treatment is financially feasible, where subclinical or clinical disease is suspected, to determine the response to treatment, or to screen horses over 15 years of age [23]. Due to a prevalence of PPID of approximately 20–25% in horses over 15 years of age, PPID should be identified as a possible causative or contributory factor for many conditions in older horses [22]. The current study demonstrates that even when no obvious hypertrichosis is present, endocrinological abnormalities may be marked. Untreated cases of PPID may result in substantial morbidity through immune dysfunction and opportunistic infections, thermoregulatory disturbances and epaxial muscle wastage which was demonstrated in these horses. The marked increases in ACTH concentrations that can be demonstrated in horses with subclinical disease emphasize the significant endocrine disturbances that can be present early in the disease process. 

Currently, there is no ante-mortem diagnostic test that can be completely relied upon for an accurate diagnosis of PPID. Measurement of basal α-MSH concentrations has shown promise in the diagnosis of early PPID [24,28,65]. Plasma α-MSH is a direct product of the pituitary PI and is secreted in greater quantities than ACTH. It has been suggested that α-MSH concentrations can increase earlier in the disease process than ACTH and may be more useful in the detection of subclinical disease compared to basal ACTH [23,28,65,66]. α-MSH also has a greater specificity and sensitivity for detection of PPID in the autumn compared to basal ACTH [24]. However, the test is not commercially available [28]. Further research into basal α-MSH is warranted to determine whether α-MSH could be superior in the detection of subclinical disease than TRH-stimulated ACTH.

## 5. Study Limitations

This is a descriptive case series, using a small sample size of only seven, light-breed horses located in Queensland Australia. While most horses were tested most months over the three and a half hear period, some ACTH concentration and hypertrichosis score data points are missing. Because of these factors, statistical significance of results could not be established. Further large-scale studies are required to gain a greater understanding of transitional PPID. 

Because there is no perfect ante-mortem or post-mortem diagnostic test for PPID, the diagnosis of subclinical PPID in these cases is imperfect. The gold standard diagnosis was performed at post-mortem on one horse with subtle clinical signs, which did reveal pituitary hyperplasia. However, it is impossible to determine whether this was an age-related change and inter-observer variation between pathologists exists [67]. 

A novel, detailed hypertrichosis scoring system was created, but intra and interobserver differences between a number of scorers would be required for validation. Therefore, the hypertrichosis scoring remains a subjective test.

## 6. Conclusions

PPID is a common disease in geriatric horses that can have a substantial impact on quality of life, and life-threatening consequences. Hypertrichosis is pathognomonic for PPID but horses with few to no clinical signs can still have substantial endocrinological disturbances. The presence of subtle signs such as recurrent infections, weight loss, reduced epaxial musculature and delayed healing in geriatric horses should raise suspicion of PPID even when pathognomonic hypertrichosis is not present. Laboratory tests are recommended to diagnose PPID, but in the early stages of disease, laboratory results can be equivocal. Basal ACTH can be measured as a simple, first line diagnostic technique for diagnosis of PPID, and is recommended in horses with severe, clinical disease. This study showed no benefit of using the TRH-stimulation test over basal ACTH in horses with severe end-stage disease. However, in early disease, the TRH-stimulation test substantially increased accuracy of diagnosis, particularly in late summer and autumn. In cases where the TRH-stimulation test is not possible and subclinical or early PPID is suspected, a basal ACTH test can still be performed. In this study, accuracy of the basal ACTH test was improved in the late summer and autumn. If the horse owner wishes to mitigate the deleterious consequences of clinical PPID identification of sub-clinically affected horses by detection of repeatedly elevated ACTH concentrations and judicious prophylactic treatment with pergolide may be warranted. 

## Figures and Tables

**Table 1:** Hypertrichosis scoring system with grey scale used to classify severity of clinical signs. The horse pictured (Horse 2) displayed the most marked clinical signs of all included study horses.

Hypertrichosis Scoring System (0–5)		
Score	Photographic Representation	Description
0/5	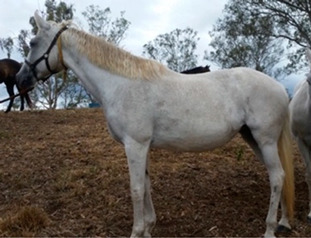	No hypertrichosis present
1/5	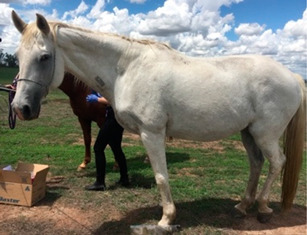	Mild hypertrichosis present, usually persistent along the ventral abdomen, palmar or plantar aspect of distal limbs, mandible and over the semimembranosus and semitendinosus muscles. Often accompanied by delayed shedding.
2/5	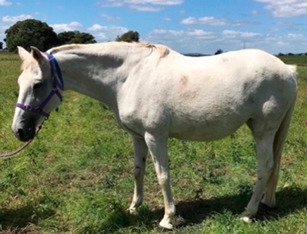	Generalized mild hypertrichosis over the entire body, accompanied by delayed shedding.
3/5	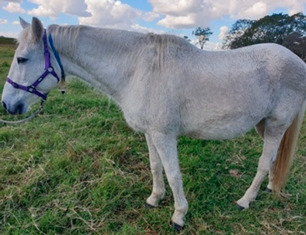	Moderate generalized hypertrichosis present over the entire body and most severe along the ventral abdomen and semimembranosus and semitendinosus muscles. Always accompanied by delayed shedding.
4/5	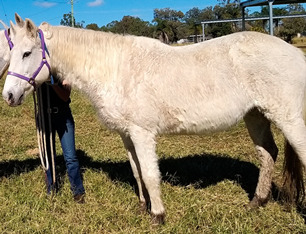	Severe generalized hypertrichosis, always accompanied by delayed shedding.
**5/5**	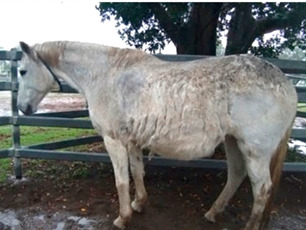	Extreme generalized hypertrichosis. Always accompanied by delayed shedding and often accompanied by difficulty thermoregulating in warmer months.

**Table 2:** Hypertrichosis score and interpretation of basal and TRH-stimulated ACTH concentrations based on DCOV.

Horse	Month/Year	Hypertrichosis Score	Basal ACTH (Likely Positive or Negative for PPID) *	TRH-Stimulated ACTH (Likely Positive or Negative for PPID) *
Horse 1	February 2019	0/5	Positive	Positive
	September 2019	1/5	Negative	Negative
	January 2020	0/5	Positive	Positive
	March 2020	0/5	Positive	Positive
Horse 2	February 2019	0/5	Positive	Positive
	September 2019	5/5	Positive	Positive
	January 2020	1/5	Positive	Positive
	March 2020	2/5	Positive	Positive
	September 2020	5/5	−	−
	February 2021	2/5	Positive	Positive
	March 2021	3/5	Positive	Positive
	April 2021	3/5	Positive	Positive
	May 2021	3/5	Positive	Positive
	June 2021	4/5	Positive	−
	November 2021	2/5	Positive	Positive
Horse 3	February 2019	0/5	Positive	Positive
	March 2019	1/5	Negative	Positive
	January 2020	0/5	Positive	Positive
	September 2020	1/5	−	−
	March 2021	0/5	Negative	Negative
	April 2021	0/5	Positive	Positive
	May 2021	0/5	Negative	Positive
	June 2021	0/5	Positive	Positive
	October 2021	1/5	Positive	Positive
Horse 4	February 2019	0/5	−	−
	September 2019	2/5	Negative	Positive
	January 2020	0/5	-	-
	March 2020	0/5	Positive	Positive
	September 2020 ^1^	5/5^1^	−	−
	February 2021 ^1^	0/5 ^1^	Negative ^1^	Negative ^1^
	March 2021 ^1^	1/5 ^1^	Negative ^1^	Negative ^1^
	April 2021 ^1^	1/5 ^1^	Negative ^1^	Negative ^1^
	June 2021 ^1^	2/5	Negative ^1^	Positive ^1^
	September 2021 ^1^	3/5	−	−
	November 2021 ^1^	2/5	Positive ^1^	Positive ^1^
Horse 5	February 2019	0/5	Negative	Negative
	March 2019	0/5	Negative	Negative
	September 2019	0/5	Positive	Positive
	January 2020	0/5	−	−
	March 2020	0/5	Negative	Negative
	September 2020	0/5	−	−
	February 2021	0/5	Negative	Negative
	March 2021	0/5	Negative	Positive
	April 2021	0/5	Positive	Negative
	May 2021	0/5	Negative	Positive
	June 2021	0/5	Negative	Positive
	November 2021	0/5	Positive	Positive
Horse 6	February 2019	0/5	Negative	Positive
	September 2019	0/5	Negative	Positive
	March 2020	0/5	Negative	Positive
	February 2021	0/5	Positive	Positive
	March 2021	0/5	Negative	Positive
	April 2021	0/5	Negative	Negative
	May 2021	0/5	Negative	Positive
	September 2021	1/5	−	−
	November 2021	0/5	Negative	Negative
Horse 7	March 2019	0/5	Negative	Positive
	September 2019	1/5	Negative	Positive
	March 2020	0/5	Negative	Positive
	February 2021	0/5	Positive	Negative
	March 2021	0/5	Positive	Positive
	April 2021	0/5	Negative	Positive
	May 2021	0/5	Negative	Negative
	June 2021	0/5	Negative	Negative
	July 2021 ^2^	− ^2^	− ^2^	− ^2^

* Based on DCOV [2]. ^1^ Receiving treatment with pergolide. ^2^ Horse was subjected to euthanasia due to chronic tooth root abscessation. Histopathologic examination of the pituitary gland revealed mild, diffuse pituitary PI grade two hyperplasia, consistent with PPID.

## Data Availability

Data are contained within the article or supplementary material.

## Supplementary Materials

The following are available online at https://www.mdpi.com/article/10.3390/vetsci9100572/s1, Video S1: Respiratory distress, nostril flaring, and tachypnoea demonstrated by a horse with PPID.
